# Serum levels of IFN-γ and NGF as potential biomarkers of depressive disorders

**DOI:** 10.1017/neu.2026.10079

**Published:** 2026-06-25

**Authors:** Lei Yi, Chuanqin Liu, Wei Lin, Ni Duan

**Affiliations:** 1 The Third Department, Qingdao Mental Health Center, Qingdao, Shandong, China; 2 Department of Sleep Medicine, Qingdao Mental Health Center, Qingdao, Shandong, China

**Keywords:** depressive disorder, biomarkers, diagnosis, immune response, neurotrophic factors

## Abstract

**Background:**

Depressive disorder (DD) is a widespread mental illness that lacks objective diagnostic biomarkers, complicating early detection and personalised treatment. This study investigated the diagnostic value of serum interferon-gamma (IFN-γ), nerve growth factor (NGF), and their ratio, alongside thyroid peroxidase antibody (TPOAb) and glial fibrillary acidic protein (GFAP), in patients with DD compared to healthy controls.

**Methods:**

A total of 238 participants (118 with DD and 120 controls) were enrolled. Depression severity was assessed using DSM-5 and HAM-D criteria. Serum biomarkers were measured using enzyme-linked immunosorbent assays (ELISA), and receiver operating characteristic (ROC) analysis was performed to assess diagnostic performance.

**Results:**

DD patients exhibited significantly lower IFN-γ and higher NGF levels than controls (both *p* < 0.001), resulting in a markedly reduced IFN-γ/NGF ratio. The IFN-γ/NGF ratio achieved the highest diagnostic accuracy (AUC = 0.858, sensitivity = 82.20%, specificity = 77.50%), outperforming IFN-γ (AUC = 0.766) and NGF (AUC = 0.848) alone. TPOAb and GFAP levels did not differ significantly between groups.

**Conclusion:**

The IFN-γ/NGF ratio is a promising biomarker for depressive disorder, offering superior diagnostic accuracy over individual immune or neurotrophic markers. This composite index may support more objective and biologically informed diagnosis in clinical psychiatry.

## Graphical abstract

**Figure:**
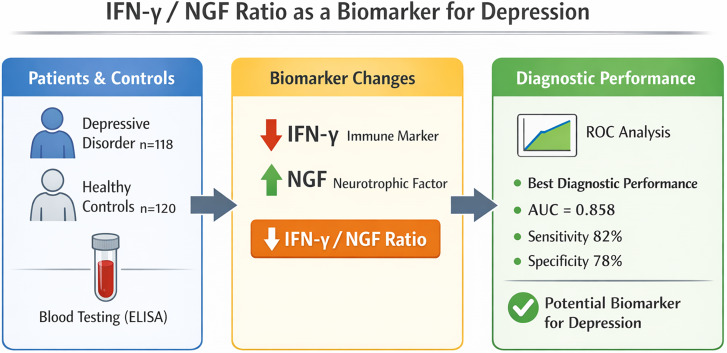

Significant outcomes

- The IFN-γ/NGF ratio achieved an excellent Area Under the Curve (AUC) of 0.858 for distinguishing DD patients from healthy controls, with high sensitivity (82.20%) and specificity (77.50%). Its diagnostic efficacy significantly outperformed the use of IFN-γ or NGF alone.
- The results confirm a significant immune-neuroendocrine disturbance in DD patients, characterised by downregulated IFN-γ and upregulated NGF levels, providing crucial new insights into the disorder’s biology.

Limitations

- The case-control design cannot determine whether the observed biomarker alterations are a cause or a consequence of depressive disorder. Additionally, the one-week medication washout period may not fully eliminate residual pharmacological effects on biomarker levels; future studies in drug-naïve populations are warranted.

## Introduction

Depressive disorder (DD) is one of the most prevalent mental health conditions globally, affecting over 280 million people and contributing significantly to the global burden of disease (Marx *et al*., 2023). Characterised by persistent sadness, anhedonia, cognitive impairments, and functional disability, DD is not only a leading cause of years lived with disability (YLDs) but also a major contributor to premature mortality through suicide and comorbid medical illnesses (Cui *et al*., 2024). Despite the widespread use of standardised diagnostic criteria such as the DSM-5 and structured clinical interviews, diagnosis remains largely reliant on subjective assessments of mood and behaviour, which can be inconsistent, prone to bias, and limited in predictive value (Khan *et al*., 2021; Marx *et al*., 2023; Spytska, 2024).

A critical clinical gap lies in the absence of objective, reliable, and reproducible biological markers for the diagnosis and stratification of depression. This lack of biomarkers impairs early detection, delays effective intervention, and hinders efforts to personalise treatment strategies (Malik *et al*., 2021; Winter *et al*., 2024). Patients often experience prolonged periods of untreated or inadequately treated illness, which is associated with poorer long-term outcomes, greater risk of relapse, and increased healthcare costs (Kuchimova, 2021). Moreover, existing diagnostic tools offer limited ability to differentiate depression from other neuropsychiatric or somatic conditions with overlapping symptoms, such as anxiety disorders, chronic fatigue, or thyroid dysfunction (Dubovsky *et al*., 2021).

Emerging research has highlighted the role of immune and neurotrophic dysregulation in the aetiology of depression. Proinflammatory cytokines such as interferon-gamma (IFN-γ) have been shown to disrupt neurotransmitter metabolism, neuroendocrine function, and neuroplasticity, contributing to the onset and persistence of depressive symptoms (Lai *et al*., 2023). Conversely, neurotrophic factors such as nerve growth factor (NGF) play a vital role in maintaining neuronal health, and their deficiency has been linked to neurodegenerative changes observed in depression (Jaiswal *et al*., 2023; Salsabil *et al*., 2023). However, these findings are often reported in isolation and have not been adequately translated into diagnostic frameworks.

Importantly, there is an urgent need to integrate immune and neurotrophic markers into a composite diagnostic model. The IFN-γ/NGF ratio may serve as a promising index that captures the balance between systemic inflammation and neuroprotective capacity. Additionally, other serum indicators such as thyroid peroxidase antibody (TPOAb) (Yang *et al*., 2023) and glial fibrillary acidic protein (GFAP) (Michel *et al*., 2021), markers of autoimmune and glial dysfunction respectively, may provide further insight into the heterogeneity of depressive pathology.

This study was undertaken to address the pressing need for objective biomarkers in the diagnosis of depressive disorders. By comparing serum IFN-γ, NGF, TPOAb, and GFAP levels in patients with depressive disorders and matched healthy controls, and evaluating their diagnostic performance – individually and in combination – this work aims to establish a foundation for biologically informed clinical tools. Such tools have the potential to improve early detection, reduce diagnostic uncertainty, and ultimately enhance treatment outcomes for individuals affected by depression.

## Methods

### Study design and participant recruitment

This case-control study aimed to investigate immunological and neurotrophic biomarkers in patients with DD compared to healthy individuals. In this study, all patients with depression had experienced depressive symptoms for at least two weeks. The exclusion criteria were as follows: a history of alcohol or illegal drug abuse or substance dependence within the past 6 months, kidney disease or inflammatory disease, heart disease, and acute/chronic infection. Since antidepressants and antipsychotics can affect the levels of various substances in the body, all participants were required to discontinue the use of these medications for at least one week prior to the test. This study involved DD patients and sex- and age-matched healthy controls. Compliance with the washout requirement was confirmed through participant self-report and review of medical records where available.

Prior to the initiation of the trial, each participant was evaluated by a qualified psychiatrist. The screening procedures included psychiatric interviews; standardised diagnostic consultations based on the Diagnostic and Statistical Manual of Mental Disorders, Fifth Edition (DSM-5); assessment of depression severity using the Hamilton Depression Rating Scale (HAM-D); evaluation of past mental health issues; and collection of sociodemographic information via pre-designed questionnaires.

**Figure 1.**
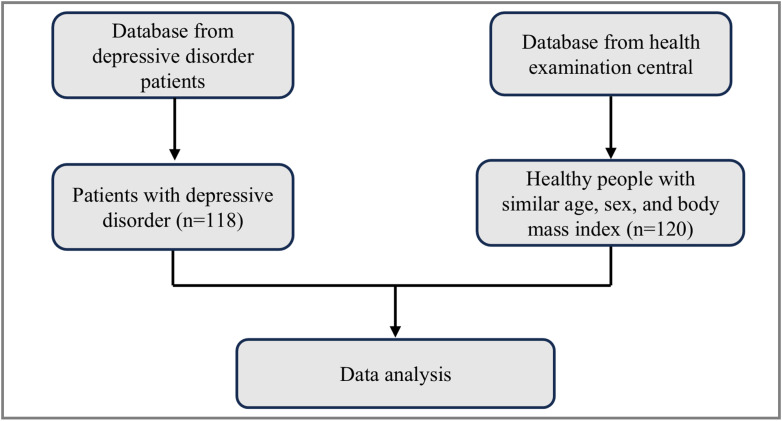
Flow chart of the study participants.

Participants were recruited from two independent sources: individuals with clinically diagnosed depressive disorders were identified through a psychiatric outpatient clinic database, while healthy controls were selected from the health examination centre of the same institution. The flow of participant inclusion is illustrated in Figure 1.

Inclusion criteria for the DD group included: (1) diagnosis of major depressive disorder in accordance with DSM-5 criteria, (2) age between 18 and 60 years, and (3) no major physical or neurological illness. Healthy controls were matched for age and sex, with no history of psychiatric disorders.

The study was approved by Qingdao Mental Health Center (#2023-V82-03). This study was conducted in accordance with the Declaration of Helsinki. We provided each participant with a detailed explanation of the study’s purpose and objectives and obtained their written informed consent prior to their participation. If a participant was found to have an intellectual disability, consent was obtained from their legal guardian. Furthermore, the detailed information of all patients was de-identified to ensure that they could not be identified in any manner.

### Sociodemographic and clinical data collection

Sociodemographic data such as age, sex, marital status, education level, smoking status, economic status, area of residence, and BMI were collected through structured interviews and medical records. Clinical history regarding previous or familial depressive disorders was also documented. Depression severity was assessed using the DSM-5 criteria and Hamilton Depression Rating Scale (HAM-D) (Renemane & Vrublevska, 2021).

### Blood sample collection and biomarker analysis

Venous blood samples were collected from all participants between 8:00 and 10:00 AM after an overnight fast. Serum was separated and stored at −80°C until analysis. Concentrations of interferon-gamma (IFN-γ), NGF, TPOAb, and GFAP were quantified using enzyme-linked immunosorbent assay (ELISA) kits following the manufacturer’s instructions.

### Statistical analysis

Data were processed and examined using IBM SPSS Statistics for Windows, version 25.0 (IBM Corp., Armonk, NY, USA). Continuous variables were expressed as mean ± standard error of the mean (SEM), and categorical variables as frequencies and percentages. Comparisons between groups were performed using the Student’s *t*-test for continuous variables and the chi-square test for categorical variables. ROC curve analysis was conducted to evaluate the diagnostic performance of IFN-γ, NGF, and their ratio. A *p*-value of less than 0.05 was considered statistically significant.

## Results

### Participant recruitment and demographics

A total of 238 participants were included in the final analysis, with 118 individuals diagnosed with DD and 120 healthy controls. Figure 1 depicts the recruitment flowchart detailing inclusion from respective databases.

As shown in Table 1, there were no statistically significant differences in age (33.23 ± 0.98 vs. 32.46 ± 0.84 years, *p* = 0.053), sex distribution (*p* = 0.435), marital status (*p* = 0.616), BMI (*p* = 0.061), education level (*p* = 0.771), economic condition (*p* = 0.630), smoking status (*p* = 0.194), or area of residence (*p* = 0.528) between the two groups. However, prior personal history of depression (71.2% vs. 0%, *p* < 0.001) and family history of depressive disorders (31.4% vs. 1.7%, *p* < 0.001) were significantly more common in the DD group.

**Table 1.** Sociodemographic characteristics of the study population

Characteristics	DD patients (*n* = 118)	Healthy controls (*n* = 120)	*p*-Value
Age (years)	33.23 ± 0.98	32.46 ± 0.84	0.053
18–24	28 (23.7%)	11 (9.1%)	
25–34	42 (35.6%)	51 (42.5%)	
35–44	34 (28.8%)	32 (26.7%)	
45–60	14 (11.9%)	26 (21.7%)	
Sex			0.435
Female	55 (46.6%)	62 (51.7%)	
Male	63 (53.4%)	58 (48.3%)	
Marital status			0.616
Married	71 (60.2%)	76 (63.3%)	
Unmarried	47 (39.8%)	44 (36.7%)	
BMI (kg/m^2^)	23.33 ± 0.52	23.46 ± 0.46	0.061
<18.5 (CED)	7 (5.9%)	4 (3.3%)	
18.5–25 (normal)	74 (62.7%)	77 (64.2%)	
>25 (obese)	37 (31.4%)	39 (32.5%)	
Education level			0.771
Illiterate	3 (2.5%)	5 (4.2%)	
Primary level	35 (29.7%)	38 (31.7%)	
Secondary level	52 (44.1%)	46 (38.3%)	
Graduate and above	28 (23.7%)	31 (25.8%)	
Economic condition			0.630
High	15 (12.7%)	18 (15.0%)	
Medium	62 (52.5%)	67 (55.8%)	
Low	41 (34.8)	35 (29.2%)	
Smoking status			0.194
Smoker	12 (10.2%)	19 (15.8%)	
Non-smoker	106 (89.8%)	101 (84.2%)	
Area of residence			0.528
Rural	32 (27.1%)	37 (30.8%)	
Urban	86 (72.9%)	83 (69.2%)	
Previous history of DD			<0.001
Yes	84 (71.2%)	0 (0%)	
No	34 (28.8%)	120 (100%)	
Family history of DD			<0.001
Yes	37 (31.4%)	2 (1.7%)	
No	81 (68.6%)	118 (98.3%)	

Data are presented as mean ± SD or *n* (%). Values of *p* < 0.05 were considered statistically significant. BMI, body mass index; CED, chronic energy deficiency; DD, depressive disorder.

### Altered immune and neurotrophic biomarkers in depressive disorder

Table 2 summarises the clinical features and laboratory parameters for both groups. DD patients exhibited significantly elevated scores on both the DSM-5 (7.57 ± 0.12 vs. 2.06 ± 0.13, *p* < 0.001) and HAM-D (17.25 ± 0.33 vs. 3.24 ± 0.22, *p* < 0.001) scales compared to controls.

**Table 2.** Clinical features and laboratory findings of study participants

Parameters	DD patients (*n* = 118)	Healthy controls (*n* = 120)	*p*-Value
DSM-5 score	7.57 ± 0.12	2.06 ± 0.13	<0.001
HAM-D score	17.25 ± 0.33	3.24 ± 0.22	<0.001
Serum IFN-γ level (pg/mL)	4.43 ± 2.45	7.42 ± 3.25	<0.001
Serum NGF level (pg/mL)	109.11 ± 31.09	73.44 ± 17.38	<0.001
IFN-γ/NGF ratio	0.05 ± 0.08	0.11 ± 0.06	<0.001
TPOAb level (IU/mL)	21.22 ± 5.26	19.95 ± 7.23	0.122
Serum GFAP level (ng/mL)	0.35 ± 0.13	0.38 ± 0.17	0.533

Data are presented as mean ± SD. Values of *p* < 0.05 were considered statistically significant.

**Figure 2.**
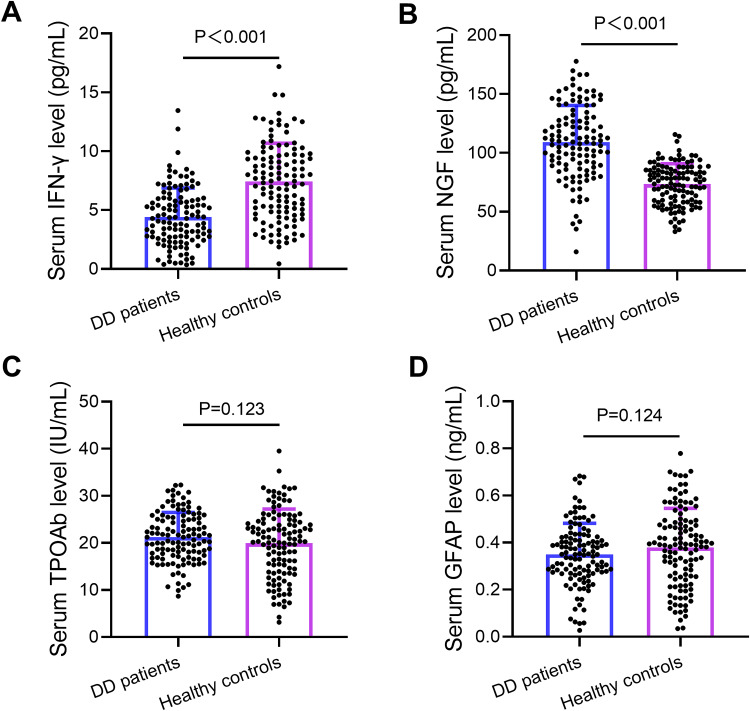
Variations of serum IFN-γ (A), NGF (B), TPOAb (C) and GFAP (D) among the study population.

Notably, IFN-γ levels were significantly decreased in DD patients (4.43 ± 2.45 pg/mL) versus healthy individuals (7.42 ± 3.52 pg/mL, *p* < 0.001), whereas NGF levels were significantly elevated (109.11 ± 31.09 pg/mL vs. 73.44 ± 17.38 pg/mL, *p* < 0.001). Consequently, the IFN-γ/NGF ratio was significantly lower in the DD group (0.05 ± 0.08) than in controls (0.11 ± 0.06, *p* < 0.001). There were no significant group differences in TPOAb or GFAP levels (both *p* > 0.1). Figure 2A–2D illustrates these biomarker variations graphically.

### IFN-γ/NGF ratio demonstrates high diagnostic accuracy for depressive disorder

**Figure 3.**
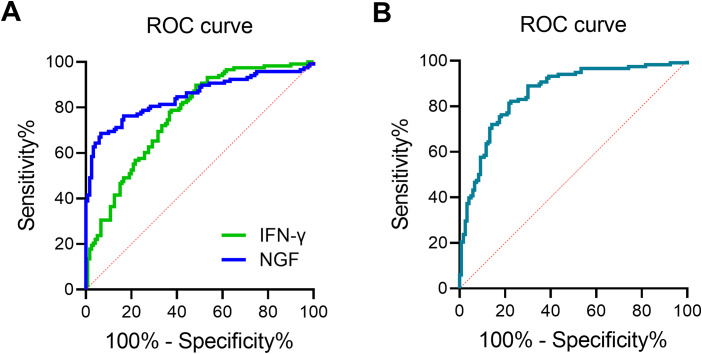
ROC curves for the healthy controls group and the DD patient group and the combined auxiliary diagnostic models. (A) ROC curves for IFN-γ and NGF. (B) ROC curve for IFN-γ/NGF ratio. The model was established based on the IFN-γ/NGF ratio of each sample and whether the subject was a DD patient.

Receiver operating characteristic (ROC) analysis was performed to evaluate the diagnostic performance of individual and combined biomarkers (Figure 3). IFN-γ alone yielded an AUC of 0.766 (95% CI: 0.707–0.818, *p* < 0.001) with a sensitivity of 89.83% and specificity of 51.67% at a cut-off value of 7.27 pg/mL (Figure 3A). NGF also demonstrated strong diagnostic performance (AUC = 0.848, 95% CI: 0.796–0.891, *p < 0.001*) with a sensitivity of 68.64% and specificity of 93.33% at a cut-off of 95.56 pg/mL (Figure 3A).

In contrast, the IFN-γ/NGF ratio demonstrated the highest diagnostic performance, with an AUC of 0.858 (95% CI: 0.808–0.900, *p* < 0.001), sensitivity of 82.20%, and specificity of 77.50% at a cut-off of 0.06 (Figure 3B). These findings underscore the ratio’s potential as a robust auxiliary marker for the clinical assessment of depressive disorder.

## Discussion

This study provides novel evidence that immune and neurotrophic biomarkers – particularly the IFN-γ/NGF ratio – may serve as valuable adjuncts to the clinical diagnosis of DD. Compared to healthy controls, individuals with DD exhibited significantly lower levels of IFN-γ and elevated levels of NGF, resulting in a markedly reduced IFN-γ/NGF ratio. Among all tested parameters, this ratio demonstrated the highest diagnostic accuracy, with strong sensitivity and specificity, underscoring its potential clinical relevance.

Our findings both align with and expand upon prior work examining cytokine and neurotrophic factor alterations in depression (Gadad *et al*., 2021; Horita *et al*., 2021). The observed reduction in IFN-γ may reflect a state of immune suppression often seen in chronic or recurrent depressive states. This phenomenon may be linked to chronic stress, dysregulation of the HPA axis, and impaired cellular immune responses (Lei *et al*., 2025). The elevated NGF levels observed in the DD group may represent a compensatory neurotrophic response to neuronal stress or damage. Our findings are consistent with models suggesting that neurotrophic systems may upregulate in response to prolonged pathological changes as a form of endogenous neuroprotection (Valencia-Sanchez *et al*., 2021). This finding appears to contrast with the widely reported inflammatory hypothesis of depression, which typically associates IFN-γ with elevated pro-inflammatory activity; however, several explanations may account for this discrepancy. First, illness stage appears critical: while acute or early-stage depression may involve heightened immune activation, chronic or recurrent illness may lead to immune exhaustion and paradoxical cytokine suppression. Second, although a one-week washout was implemented, residual immunomodulatory effects of prior pharmacotherapy cannot be entirely excluded, as certain antidepressants can durably alter cytokine profiles beyond the washout window. Third, the findings may reflect a Th1-to-Th2 immune shift, in which declining Th1-driven IFN-γ production accompanies a relative rise in Th2-mediated responses – a pattern documented in specific subsets of depressed patients in prior research. Notably, similar IFN-γ reductions have been reported in patients with recurrent or treatment-resistant depression, suggesting that the present results may represent a biologically distinct depression phenotype rather than a contradiction of the inflammatory model. It is important to acknowledge, however, that NGF findings in the depression literature are heterogeneous: while the present study and certain prior reports observe elevated NGF, other studies have found decreased or unchanged levels in depressed populations (Jaiswal *et al*., 2023; Salsabil *et al*., 2023). These divergent findings may reflect differences in illness stage, depression subtype, medication history, and the degree or chronicity of neuronal stress. For instance, early or mild depression may be associated with NGF suppression, whereas chronic or severe illness may trigger compensatory neurotrophic upregulation. Depression subtypes – such as melancholic or treatment-resistant forms – may also display distinct neurotrophic profiles. Future studies with clearly stratified patient subgroups will be necessary to resolve these inconsistencies and better characterise the directionality of NGF changes across the depressive spectrum.

One important contribution of our study is the evaluation of the IFN-γ/NGF ratio as a combined marker, rather than interpreting these biomarkers independently. This integrative and novel approach captures the dynamic interaction between inflammatory and neurotrophic pathways, providing a more comprehensive biological signal that is more diagnostically robust than either marker alone. The strong discriminative performance of this ratio suggests it could be a reliable auxiliary diagnostic tool in clinical psychiatry.

In contrast, serum levels of TPOAb and GFAP did not significantly differ between groups. These findings suggest that autoimmune thyroid dysfunction and astrocytic activation may not be central to the pathophysiology of depression in this specific cohort, which has recently been an active research area (Sawicka-Gutaj *et al*., 2022; Lekurwale *et al*., 2023). It is also possible that these markers are only altered in specific subtypes or severities of depression not represented here.

The clinical implications of these results are significant. The integration of objective, blood-based biomarkers into routine psychiatric assessment could reduce reliance on subjective symptom reporting, decrease diagnostic uncertainty, and improve early intervention. Particularly in primary care or resource-limited settings, such biomarkers could serve as accessible and efficient tools for identifying at-risk individuals and guiding personalised treatment decisions.

Nonetheless, several limitations should be noted. The cross-sectional nature of the study precludes causal interpretation and limits the ability to assess biomarker changes over time. The relatively homogeneous demographic characteristics of the sample may also restrict generalisability. Furthermore, factors such as medication use, dietary habits, sleep quality, and comorbid physical conditions were not exhaustively controlled, and these may influence biomarker levels. Moreover, the one-week medication washout period, while intended to reduce pharmacological confounding, may not fully eliminate residual biological effects of prior antidepressant or antipsychotic treatment, particularly for agents with extended half-lives or sustained immunomodulatory properties. Future investigations employing drug-naïve patient cohorts would be valuable to confirm these biomarker findings in a fully unmedicated state.

Future research should employ longitudinal study designs to evaluate biomarker dynamics across different phases of illness and treatment. Investigations that include larger, more diverse populations and incorporate additional biological and imaging markers could further enhance the diagnostic specificity and mechanistic understanding of depression. Exploring the utility of the IFN-γ/NGF ratio in differentiating depression from other neuropsychiatric or inflammatory conditions may also extend its clinical utility.

## Conclusion

In summary, this study highlights the IFN-γ/NGF ratio as a promising biomarker with high diagnostic performance in depressive disorder. By reflecting the interplay between immune and neurotrophic processes, this ratio offers a biologically meaningful tool that could facilitate earlier and more precise identification of individuals with depression, ultimately improving clinical outcomes and supporting personalised mental health care.

## Data Availability

The data used to support the findings of this study are available from the corresponding author upon request.

## References

[ref1] Cui L , Li S , Wang S , Wu X , Liu Y , Yu W , Wang Y , Tang Y , Xia M and Li B (2024) Major depressive disorder: Hypothesis, mechanism, prevention and treatment. Signal Transduction and Targeted Therapy 9, 30. 38331979 10.1038/s41392-024-01738-y PMC10853571

[ref2] Dubovsky SL , Ghosh BM , Serotte JC and Cranwell V (2021) Psychotic depression: Diagnosis, differential diagnosis, and treatment. Psychotherapy and Psychosomatics 90, 160–177. 10.1159/000511348. 33166960

[ref3] Gadad BS , Vargas-Medrano J , Ramos EI , Najera K , Fagan M , Forero A and Thompson PM (2021) Altered levels of interleukins and neurotrophic growth factors in mood disorders and suicidality: An analysis from periphery to central nervous system. Translational Psychiatry 11, 341. 10.1038/s41398-021-01452-1. 34078872 PMC8171230

[ref4] Horita JKHA , da Silva MCM , Ferrari CZ , Vieira ELM , Moreira FA , de Oliveira ACP and Reis HJ (2021) Evaluation of brain cytokines and the level of brain-derived neurotrophic factor in an inflammatory model of depression. Neuroimmunomodulation 27, 87–96. 10.1159/000511181 33176302

[ref5] Jaiswal A , Shreekantiah U and Goyal N (2023) Nerve growth factor in psychiatric disorders: A scoping review. Indian Journal of Psychological Medicine 45, 555–564. 10.1177/02537176231162518. 38545533 PMC10964880

[ref6] Khan DM , Yahya N , Kamel N and Faye I (2021) Automated diagnosis of major depressive disorder using brain effective connectivity and 3D convolutional neural network. IEEE Access 9, 8835–8846.

[ref7] Kuchimova CA (2021) Clinical features of prolonged depression and differentiated treatment options. In: Наука и прогресс: время перемен. pp 140–144.

[ref8] Lai JY , Ho JX , Kow ASF , Liang G , Tham CL , Ho YC , Lee MT (2023) Interferon therapy and its association with depressive disorders – A review. Frontiers in Immunology 14, 1048592. 10.3389/fimmu.2023.1048592. 36911685 PMC9992192

[ref9] Lei AA , Phang VWX , Lee YZ , Kow ASF , Tham CL , Ho YC , Lee MT (2025) Chronic stress-associated depressive disorders: The impact of HPA axis dysregulation and neuroinflammation on the hippocampus -A mini review. International Journal of Molecular Sciences 26, 2940. 10.3390/ijms26072940. 40243556 PMC11988747

[ref10] Lekurwale V , Acharya S , Shukla S and Kumar S (2023) Neuropsychiatric manifestations of thyroid diseases. Cureus 15, e33987. 10.7759/cureus.33987. 36811059 PMC9938951

[ref11] Malik S , Singh R , Arora G , Dangol A and Goyal S (2021) Biomarkers of major depressive disorder: Knowing is half the battle. Clinical Psychopharmacology and Neuroscience 19, 12. 33508785 10.9758/cpn.2021.19.1.12 PMC7851463

[ref12] Marx W , Penninx BW , Solmi M , Furukawa TA , Firth J , Carvalho AF , Berk M (2023) Major depressive disorder. Nature Reviews Disease Primers 9, 44. 10.1038/s41572-023-00454-1 37620370

[ref13] Michel M , Fiebich BL , Kuzior H , Meixensberger S , Berger B , Maier S , Nickel K , Runge K , Denzel D , Pankratz B , Schiele MA , Domschke K , van Elst LT and Endres D (2021) Increased GFAP concentrations in the cerebrospinal fluid of patients with unipolar depression. Translational Psychiatry 11, 308. 10.1038/s41398-021-01423-6. 34021122 PMC8139962

[ref14] Renemane L and Vrublevska J (2021) Hamilton depression rating scale: Uses and applications. In Martin CR, Hunter LA and Rajendram R (eds), The neuroscience of depression. Elsevier, pp. 175–183.

[ref15] Salsabil L , Shahriar M , Islam SMA , Bhuiyan MA , Qusar MS and Islam MR (2023) Higher serum nerve growth factor levels are associated with major depressive disorder pathophysiology: A case-control study. Journal of International Medical Research 51, 3000605231166222. 10.1177/03000605231166222. 37038918 PMC10107982

[ref16] Sawicka-Gutaj N , Zawalna N , Gut P and Ruchała M (2022) Relationship between thyroid hormones and central nervous system metabolism in physiological and pathological conditions. Pharmacological Reports 74, 847–858. 35771431 10.1007/s43440-022-00377-w

[ref17] Spytska L (2024) Anxiety and depressive personality disorders in the modern world. Acta Psychologica 246, 104285. 10.1016/j.actpsy.2024.104285. 38642453

[ref18] Valencia-Sanchez C , Pittock SJ , Mead-Harvey C , Dubey D , Flanagan EP , Lopez-Chiriboga S , Trenerry MR , Zalewski NL , Zekeridou A and McKeon A (2021) Brain dysfunction and thyroid antibodies: Autoimmune diagnosis and misdiagnosis. Brain Communications 3, fcaa233. 10.1093/braincomms/fcaa233. 34061124 PMC8152924

[ref19] Winter NR , Blanke J , Leenings R , Ernsting J , Fisch L , Sarink K , Barkhau C , Emden D , Thiel K , Flinkenflügel K , Winter A , Goltermann J , Meinert S , Dohm K , Repple J , Gruber M , Leehr EJ , Opel N , Grotegerd D , Redlich R , Nitsch R , Bauer J , Heindel W , Gross J , Risse B , Andlauer TFM , Forstner AJ , Nöthen MM , Rietschel M , Hofmann SG , Pfarr J-K , Teutenberg L , Usemann P , Thomas-Odenthal F , Wroblewski A , Brosch K , Stein F , Jansen A , Jamalabadi H , Alexander N , Straube B , Nenadić I , Kircher T , Dannlowski U and Hahn T (2024) A systematic evaluation of machine learning-based biomarkers for major depressive disorder. JAMA Psychiatry 81, 386–395. 10.1001/jamapsychiatry.2023.5083. 38198165 PMC10782379

[ref20] Yang W , Qu M , Jiang R , Lang X and Zhang X-Y (2023) Association between thyroid function and comorbid anxiety in first-episode and drug naïve patients with major depressive disorder. European Archives of Psychiatry and Clinical Neuroscience 273, 191–198. 35851661 10.1007/s00406-022-01457-x

